# Ventricular conduction abnormality in patients with mild to moderate cardiomyopathy

**DOI:** 10.1002/clc.24001

**Published:** 2023-03-07

**Authors:** Mehak Dhande, Konstantinos N. Aronis, Floyd Thoma, Suresh Mulukutla, Aditya Bhonsale, Krishna Kancharla, Alaa Shalaby, Andrew Voigt, N. A. Mark Estes, Sandeep K. Jain, Samir Saba

**Affiliations:** ^1^ Heart and Vascular Institute University of Pittsburgh Medical Center Pittsburgh Pennsylvania USA

**Keywords:** cardiac resynchronization therapy, heart failure, hospitalization, mild‐to‐moderate cardiomyopathy, mortality

## Abstract

**Background:**

In mild‐to‐moderate cardiomyopathy, cardiac resynchronization therapy (CRT) is indicated in patients with high burden of right ventricular pacing but not in those with intrinsic ventricular conduction abnormalities.

**Hypothesis:**

We hypothesized that CRT positively impacts outcomes of patients with intrinsic ventricular conduction delay and left ventricular ejection fraction (LVEF) of 36%‐50%.

**Methods:**

Of 18 003 patients with LVEF ≤ 50%, 5966 (33%) patients had mild‐to‐moderate cardiomyopathy, of whom 1741 (29%) have a QRS duration ≥120 ms. Patients were followed to the endpoints of death and heart failure (HF) hospitalization. Outcomes were compared between patients with narrow versus wide QRS.

**Results:**

Of the 1741 patients with mild‐to‐moderate cardiomyopathy and wide QRS duration, only 68 (4%) were implanted with a CRT device. Over a median follow‐up of 3.35 years, 849 (51%) died and 1004 (58%) had a HF hospitalization. The adjusted risk of death (hazard ratio (HR) = 1.11, *p* = 0.046) and of death or HF hospitalization (HR = 1.10, *p* = 0.037) were significantly higher in patients with wide versus narrow QRS duration. In patients with wide QRS complex, CRT was associated with reduction in the adjusted risk of death (HR = 0.47, *p* = 0.020) and of death or HF hospitalization (HR = 0.58, *p* = 0.008).

**Conclusions:**

Patients with mild‐to‐moderate cardiomyopathy and wide QRS duration are rarely implanted with CRT devices and have worse outcomes compared to those with narrow QRS. Randomized trials are needed to examine if CRT has salutary effects in this population.

## Graphical abstract

In patients with intermediate cardiomyopathy 35%–50% of any etiology), the presence of ventricular conduction abnormalities (QRS > 120 ms) is associated with worse mortality and composite of death or heart failure hospitalization. In these patients, the presence of cardiac resynchronization therapy device is associated with improved outcomes (M. Dhande, K.N. Aronis, F. Thoma, S. Mulukutla, A. Bhonsale, K. Kancharla, A. Shalaby, A. Voigt, N.A.M. Estes, S.K. Jain, S. Saba……. xx‐xx).

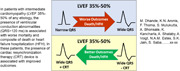

## 1 INTRODUCTION

Cardiac resynchronization therapy (CRT) is an established treatment for heart failure (HF) patients with severe cardiomyopathy and evidence of ventricular conduction abnormalities on surface electrocardiogram.^1^, ^2^, ^3^, ^4^, ^5^, ^6^ In this context, CRT provides incremental survival benefit and improvement in the symptoms of HF over established guideline directed medical therapy.^1^, ^2^, ^3^ For patients with mild‐to‐moderate cardiomyopathy whose left ventricular ejection fraction (LVEF) is between 36% and 50%, CRT is indicated only when patients are expected to have a high burden of right ventricular pacing^5^, ^7^, ^8^ but not when they exhibit evidence of intrinsic wide QRS complex on the surface electrocardiogram.^5^ It is plausible that the same mechanisms that afford benefit of CRT in severe cardiomyopathy or in the context of right ventricular pacing would also apply to patients with mild‐to‐moderate cardiomyopathy with intrinsic ventricular conduction abnormalities. To date, however, there are no clinical trials that have studies this important clinical question.

To address this gap in knowledge, we designed this clinical study using real‐world data from a large, multi‐hospital academic institution. The present analysis focuses on the impact of QRS width on mortality and HF hospitalizations in patients with mild‐to‐moderate cardiomyopathy and explores the potential role of CRT in this context.

## 2 METHODS

### 2.1 Patient population and endpoints

**Figure 1:**
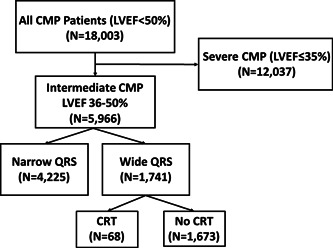
Overview of the cohort of patients with cardiomyopathy with focus on patients with mild to moderate ventricular dysfunction (LVEF 36%–50%). The cohort was stratified by QRS duration and the presence or absence of cardiac resynchronization therapy device. CMP, cardiomyopathy; CRT, cardiac resynchronization therapy; LVEF, left ventricular ejection fraction.

The present study was approved by the institutional review board of the University of Pittsburgh who waived the requirement to obtain a consent from patients, due to the retrospective, observational nature of this analysis. We performed a cohort analysis of 18 003 consecutive patients with cardiomyopathy (LVEF ≤ 50%) who were seen at the hospitals and clinics of the University of Pittsburgh Medical Center from January 1, 2011 through December 31, 2017. Of this group, 5966 (33%) had a LVEF between 36% and 50% inclusively, with 1741 patients in this group having evidence of prolonged QRS duration (≥120 ms) on surface electrocardiogram. In that latter group, only 68 patients received a CRT device. The composition of the overall data set by severity of cardiomyopathy, QRS width and CRT status is shown in Figure 1.

Baseline demographic, clinical, and medications data were obtained from the institutional electronic health and administrative records from the hospitals and outpatient clinics of the UPMC healthcare system and were compiled by the analytic warehouse for analysis. Patients were followed from the time of first documented LVEF in the 36%–50% range to the endpoints of all‐cause mortality or the composite endpoint of death or HF hospitalization, through March of 2019.

### 2.2 Statistical analysis

Baseline characteristics are presented as mean ± standard deviation for continuous variables and numbers (%) for discrete variables and compared in the cohort of patients with mild‐to moderate cardiomyopathy between those with narrow versus wide QRS duration, and in that latter group between patients with versus without CRT device, using Student *t*‐test and *χ* ^2^ test, as appropriate. Kaplan–Meier survival curves were constructed to compare the overall survival as well as the survival free from HF hospitalization between mild‐to‐moderate cardiomyopathy patients with narrow versus wide QRS and in the latter group between CRT recipients versus those who did not receive resynchronization therapy. Cox multivariable models adjusting for the age of patients at initial contact within our institution, sex, race, their type of cardiomyopathy (ischemic vs. nonischemic), and the presence of a history of hypertension, diabetes mellitus, congestive heart failure, atrial fibrillation, or liver cirrhosis in addition to the baseline serum hemoglobin level and glomerular filtration rate, were developed to examine the independent impact of QRS duration on the study endpoints as well as the impact of CRT device implantation on these same endpoints. Given the small number of CRT recipients (*n* = 68), we limited the follow‐up duration for the CRT analysis to 2 years and imputed the missing values for the serum hemoglobin and glomerular filtration rate to the mean value for these covariates. A two‐sided *p* value <0.05 was considered statistically significant. Statistical analyses were performed on SPSS software (version 27, IBM).

## 3 RESULTS

Table 1 details the baseline characteristics of the overall cohort of patients with mild‐to‐moderate cardiomyopathy, stratified by QRS width. It also details the baseline characteristics of patients with mild‐to‐moderate cardiomyopathy and prolonged QRS duration by their CRT status. As depicted in Tables 1, 1741 (33%) patients with mild‐to‐moderate cardiomyopathy had a prolonged ventricular activation time on the surface electrocardiogram and of those, only 68 patients were implanted with a CRT device. Compared to patients with a narrow QRS complex, those with prolonged QRS duration were significantly older, more likely to be white men, more likely to have coronary artery disease, heart failure, atrial fibrillation, and had more comorbidities, including hypertension, diabetes mellitus and chronic kidney disease. Within the cohort of patients with ventricular conduction delay, CRT recipients had more heart failure and atrial fibrillation but were otherwise comparable to patients who did not receive a CRT device despite having a wide QRS duration.

**Table 1:** Baseline characteristics of patients with mild‐to‐moderate cardiomyopathy stratified by QRS width and by CRT status.

	All (*N* = 5966)	QRS < 120 (*N* = 4255)	QRS ≥ 120 (*N* = 1741)	*p* Value	No CRT (*N* = 1673)	CRT (*N* = 68)	*p* Value
Age (years)	70 ± 14	68 ± 15	75 ± 12	<0.001	75 ± 12	74 ± 10	0.34
Women	37%	39%	33%	<0.001	33%	29%	0.69
Black patients	10%	11%	6%	0.033	6%	6%	0.98
BMI (Kg/m^2^)	29.4 ± 6.4	29.3 ± 7.2	29.4 ± 6.4	0.85	29.3 ± 6.4	28.4 ± 4.9	0.24
Weight (kgs)	85 ± 23	85 ± 24	86 ± 22	0.51	86 ± 22	87 ± 21	0.58
Coronary artery disease	47%	45%	52%	<0.001	51%	60%	0.17
Congestive heart failure	33%	29%	41%	<0.001	40%	71%	<0.001
Atrial fibrillation	29%	25%	37%	<0.001	36%	60%	<0.001
LVEF	40% ± 1%	40% ± 1%	40% ± 1%	0.17	40% ± 1%	40% ± 1%	0.96
QRS duration (ms)	111 ± 31	93 ± 12	150 ± 22	<0.001	150 ± 22	156 ± 19	0.025
Hypertension	61%	59%	65%	<0.001	65%	71%	0.36
Hyperlipidemia	56%	55%	59%	0.009	59%	68%	0.17
Diabetes mellitus	31%	29%	34%	<0.001	34%	35%	0.79
COPD	16%	15%	17%	0.23	17%	19%	0.62
Chronic kidney disease	10.4%	9.4%	12.4%	<0.001	12.4%	11.8%	1.00
End‐stage renal disease	2.7%	2.9%	2.2%	0.15	2.3%	1.5%	1.00
Liver cirrhosis	2.8%	3.0%	2.5%	0.38	2.5%	2.9%	0.69
Hemoglobin (g/dL)	12.2 ± 2.2	12.2 ± 2.2	12.2 ± 2.1	0.50	12.2 ± 2.1	12.4 ± 1.9	0.60
GFR (milliliter per minute)	67 ± 33	69 ± 34	64 ± 38	<0.001	64 ± 29	60 ± 23	0.34
ß‐Blockers	42%	43%	40%	0.08	40%	46%	0.38
ACEi and ARB	30%	30%	28%	0.20	28%	43%	0.013

Abbreviations: ACEi, angiotensin converting enzyme inhibitors; ARB, angiotensin receptor antagonist; BMI, body mass index; COPD, chronic obstructive pulmonary disease; CRT, cardiac resynchronization therapy; GFR, glomerular filtration rate; LVEF, left ventricular ejection fraction.

**Figure 2:**
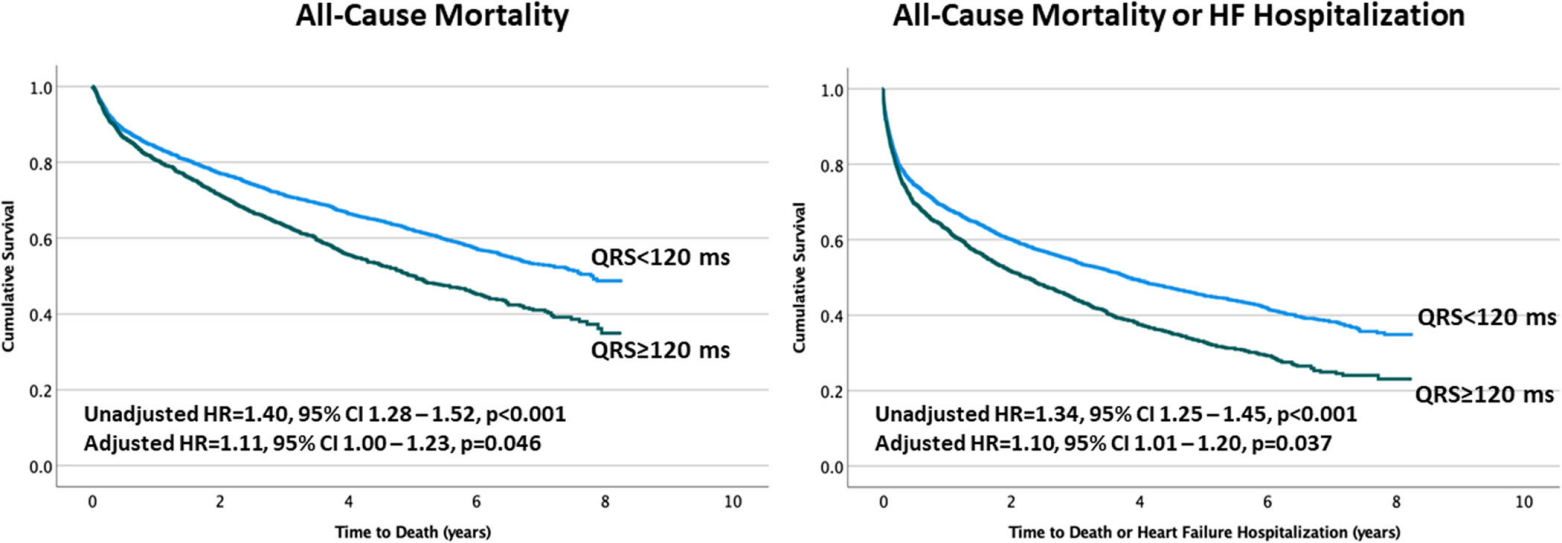
Kaplan–Meier survival curves comparing the outcome of all‐cause mortality (left panel) and the composite of all‐cause mortality or heart failure hospitalization (right panel) in patients with mild‐to‐moderate cardiomyopathy with narrow versus wide QRS duration. CI, confidence interval; HF, heart failure; HR, hazard ratio.

Over a median follow‐up of 3.35 years, 849 (51%) died and 1004 (58%) had at least one HF hospitalization. Patients with QRS ≥ 120 ms had higher all‐cause mortality (hazard ratio (HR) = 1.40, 95% confidence interval (CI) 1.28–1.52, *p* < 0.001, Figure 2) and higher risk of the composite of death or HF hospitalization (HR = 1.34, 95% CI 1.25–1.45, *p* < 0.001, Figure 2). After adjusting for unbalanced covariates, these results persisted for both the all‐cause death endpoint (HR = 1.11, 95% CI 1.00–1.23, *p* = 0.046) and the death or HF hospitalization composite endpoint (HR = 1.10, 95% CI 1.01–1.20, *p* = 0.037). Importantly, performing these same analyses while excluding the 68 patients who were implanted with a CRT device yielded similar results for the adjusted risk of death (HR = 1.12, 95% CI 1.01–1.24, *p* = 0.029) and the composite of death or HF hospitalization (HR = 1.11, 95% CI 1.01–1.21, *p* = 0.027).

**Figure 3:**
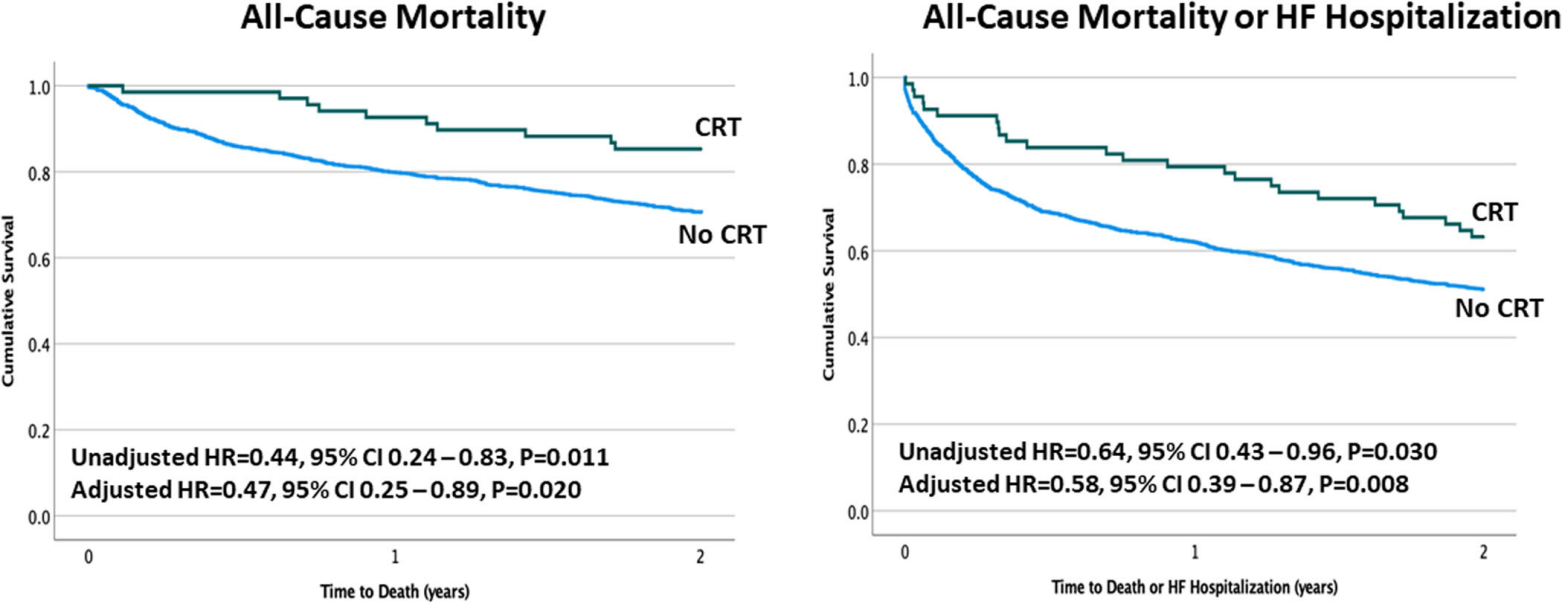
Kaplan–Meier survival curves comparing the outcome of all‐cause mortality (left panel) and the composite of all‐cause mortality or heart failure hospitalization (right panel) in patients with mild‐to‐moderate cardiomyopathy and narrow QRS duration by CRT status. CI, confidence interval; CRT, cardiac resynchronization therapy; HF, heart failure; HR, hazard ratio.

In patients with wide QRS, CRT was associated with a significant reduction in the risk of death (HR = 0.44, 95% CI 0.24–0.83, *p* = 0.011, Figure 3) and in the risk of death or HF hospitalization (HR = 0.64, 95% CI 0.43–0.96, *p* = 0.030, Figure 3). As shown in Table 2, these results remained unchanged after adjustment in the multivariable model with a relative reduction of 53% in all‐cause mortality (HR = 0.47, 95% CI 0.25–0.89, *p* = 0.020) and of 42% in the composite of death or HF hospitalizations (HR = 0.58, 95% CI 0.39–0.87, *p* = 0.008).

**Table 2:** Multivariable Cox model examining the independent predictors of the 2‐year all‐cause mortality and of the composite of death or heart failure hospitalizations in CRT recipients versus nonrecipients.

	Hazard ratio	95% Confidence interval		*p* Value
		Lower	Upper	
*All‐cause mortality*				
Cardiac resynchronization therapy	0.47	0.25	0.89	0.020
Age (per 1 year increment)	1.05	1.04	1.06	<0.001
Sex (women vs. men)	0.79	0.65	0.96	0.16
Race (Black vs. White)	0.95	0.63	1.39	0.74
Coronary artery disease	0.86	0.71	1.03	0.10
Congestive heart failure	1.12	0.93	1.34	0.24
Hypertension	0.83	0.68	1.01	0.70
Diabetes mellitus	1.38	1.15	1.67	<0.001
Atrial fibrillation	1.19	0.99	1.44	0.07
Liver cirrhosis	1.88	1.15	3.08	0.012
GFR (per 1milliliter per minute)	0.99	0.99	1.00	0.009
Hemoglobin (per 1 g/dL)	0.80	0.76	0.84	<0.001
*All‐cause mortality or heart failure hospitalization*				
Cardiac resynchronization therapy	0.58	0.39	0.87	0.008
Age (per 1 year increment)	1.03	1.03	1.04	<0.001
Sex (women vs. men)	0.98	0.85	1.14	0.82
Race (Black vs. White)	1.18	0.89	1.55	0.25
Coronary artery disease	0.97	0.84	1.12	0.68
Congestive heart failure	1.23	1.07	1.42	0.003
Hypertension	0.93	0.80	1.09	0.39
Diabetes mellitus	1.40	1.21	1.63	<0.001
Atrial fibrillation	1.23	1.07	1.42	0.004
Liver cirrhosis	2.13	1.47	3.09	<0.001
GFR (per 1milliliter per minute)	0.99	0.99	1.00	<0.001
Hemoglobin (per 1 g/dL)	0.88	0.85	0.92	<0.001

Abbreviations: CRT, cardiac resynchronization therapy; GFR, glomerular filtration rate.

We examined in detail the 68 patients with intermediate LVEF and wide QRS complex who received a CRT pacing device. In that group, 45 out of 68 patients (66%) had a LVEF ≤ 35% at the time of initial CRT implantation. These patients were included in the intermediate LVEF group in our data set because their CRT device was implanted before 2011, the date of onset of the present study, and their first documented LVEF within the study period had already improved into the intermediate range. Of note, 42 out of these 45 patients (93%) were implanted with a CRT defibrillator. Of the remaining 23 patients, all but one patient (96%) had an anticipated high burden of right ventricular pacing that is, met the Block‐HF clinical trial^7^ inclusion criteria. In this latter group, 14 out of 23 patients (61%) received a CRT pacemaker.

## 4 DISCUSSION

In the present study, we demonstrate that about one‐third of patients with left ventricular dysfunction have a mild‐to‐moderate cardiomyopathy and that one‐third of this latter group have evidence of ventricular conduction abnormalities on surface electrocardiogram. We also show a strong direct association between prolonged QRS duration and the risk of all‐cause mortality alone or in conjunction with HF hospitalizations. Importantly, despite small numbers, our data suggest that CRT may be associated with significant improvement in these endpoints, even after adjustment for unbalanced covariates. In the absence of current guideline recommendations for CRT use in the context of mild‐to‐moderate cardiomyopathy and intrinsic ventricular conduction abnormalities,^5^ our data provide the needed premise for future pivotal trials that would examine this important question and could inform guideline indications and practice patterns.

The salutary benefits of CRT in HF patients with prolonged QRS duration have been studied extensively and include improvement in measures of quality of life and exercise capacity, as well as increase in peak oxygen consumption and recovery of echocardiographic measures of contractility and reverse remodeling.^9^, ^10^ In addition, CRT has been shown to improve all‐cause mortality and the combined endpoint of death or HF events.^1^, ^2^, ^3^ The underlying mechanism of these benefits is thought to be through an increase in myocardial contractility and efficiency without a concomitant increase in oxygen consumption.^11^, ^12^ The clinical benefits of CRT have been demonstrated in the context of severe cardiomyopathy with either intrinsic ventricular conduction delay^1^, ^2^ or high burden of right ventricular pacing.^7^ Similar findings have been confirmed in patients with mild‐to‐moderate cardiomyopathy when the burden of right ventricular pacing was high.^7^, ^8^ It is therefore plausible that the same benefit would be achieved in patients with mild‐to‐moderate cardiomyopathy and intrinsic conduction abnormalities but trials examining this specific question have not been conducted. Our present data provide a strong support for the future design of such trials which may alter published CRT guidelines. Should further clinical trials reveal that CRT proves beneficial in these patients, the impact of such a guideline directed practice change would be significant as, currently, a large number of patients with intermediate cardiomyopathy and prolonged QRS duration may not undergo CRT in the absence of pacing burden criteria.

The present study has limitations. First, it is a single‐center, observational trial, which can be subject to bias. We have tried to minimize bias by including all patients from all hospitals across the system who met the inclusion criteria, without any exclusion parameters. In addition, we have applied rigorous statistical adjustments to account for any unbalanced characteristics between patient groups. However, persisting confounders may exist. Second, the present analysis lacked information about the morphology of the QRS complex on surface electrocardiogram, which has been associated with outcomes after CRT.^5^ Third, our data lacked information about follow‐up echocardiographic findings as well as the mode of death of patients. Lastly, CRT recipients in our study overwhelmingly met CRT implantation criteria according to published guidelines as opposed to being *de novo* CRT recipients in the context of intermediate LVEF and intrinsic ventricular conduction abnormality. It is very promising that patients with prior LVEF ≤ 35% who received a CRT device and improved their myocardial function, have a better prognosis than patients with LVEF 35%–50% and a wide QRS complex but without CRT. This, however, does not prove the utility of de novo CRT in patients with intermediate cardiomyopathy and wide, intrinsic QRS complex.

## 5 CONCLUSION

In summary, our study demonstrates that patients with mild‐to‐moderate cardiomyopathy and wide QRS duration are at an increased risk of death and HF hospitalization compared to those with narrow QRS. Randomized trials are needed to examine if CRT has salutary effects in this population. Results from such trials could potentially drive updates to the published CRT guidelines^5^ and afford changes in practice patterns.

## CONFLICT OF INTEREST STATEMENT

The authors declare no conflict of interest.

## Data Availability

Research data are not share.
